# Multiple endogenous seed-born bacteria recovered rice growth disruption caused by *Burkholderia glumae*

**DOI:** 10.1038/s41598-021-83794-w

**Published:** 2021-02-18

**Authors:** Chiharu Akimoto-Tomiyama

**Affiliations:** Plant and Microbial Research Unit, Division of Plant and Microbial Sciences, Institute of Agrobiological Sciences, National Agriculture and Food Research Organization, Tsukuba, Ibaraki 305-8518 Japan

**Keywords:** Biological techniques, Biotechnology, Microbiology, Plant sciences

## Abstract

*Burkholderia glumae* is a causal agent of bacterial grain and seedling rot in rice, and is a threat to stable global food supply. The virulence of *B. glumae* was suppressed when it was inoculated on budding seed rather than on non-budding seed. To clarify the phenomena, pathogen titer inside the rice plant was measured by serial dilution plating of lysates from budding rice seedlings. Surprisingly, morphologically different types of colonies were observed on the plates. These ‘contaminated’ rice seed-born bacteria (RSB) were identified by sequencing 16S rRNA genes as three strains of *Pseudomonas putida* (RSB1, RSB10, RSB15) and *Stenotrophomonas maltophilia* (RSB2). All bacteria and *B. glumae* were simultaneously inoculated onto rice seeds, and all three *P. putida* RSBs suppressed the growth disruption caused by *B. glumae*, whereas RSB2 had no effect. Thus, the virulence was synergistically suppressed when co-treated with RSBs. The effect could be dependent on the high biofilm formation ability of RSB2. By comprehensive microbiota analysis, endogenous rice flora were changed by RSBs treatment. These results suggest the possibility of novel pathogen control through pre-treatment with endogenous beneficial microorganisms. The method would contribute substantially to the implementation of sustainable agriculture stated in Sustainable Development Goals of United Nations.

## Introduction

*Burkholderia glumae* is a seed and soil-borne plant pathogen that causes bacterial seedling rot and bacterial grain rot of rice^1,2^. This is one of the most important diseases affecting rice production worldwide, particularly in East Asia, Southeast Asia, North America, and South America^3^. Because the optimal temperature range for the growth of *B. glumae* is 30–35 °C^4^, and production of toxoflavin is enhanced at high temperatures around 37 °C^5^, rapid multiplication may occur more frequently in tropical and semi-tropical countries, and the disease is likely to spread more rapidly with intense global warming. Shew et al. estimated the economic loss for a 1 °C increase in the Mid-South United States as a $112 million USD annual decrease in consumer surplus in that specific area of US, and a loss of production equivalent to feeding 2.17 million people^6^.

In Japan, most rice seeds are sown in nursery boxes, and seedlings are moved to nursery beds before transplanting to the rice field. Because nursery boxes are maintained under relatively high temperatures (28–30 °C) to promote simultaneous germination, seedling rot tends to occur in case seeds had been infected^7,8^. Sometimes apparently healthy seeds but contaminated with *B. glumae* are sown and transplanted, causing eventual development of grain rot in the field^3,9,10^. Recently, one of the measures to controlling plant disease is breeding tolerant rice varieties^11^, and some virulent *B. glumae* strains have been analyzed genetically to identify putative virulence genes. However, a rice cultivar resistant to *B. glumae* is not commercially available at this time. Other major means for the control of *B. glumae* in Japan^12^ is seed treatment with oxolinic acid, a quinoline derivative. However, the occurrence of strains naturally resistant to oxolinic acid has been a serious limitation to this method of disease control^12,13^.

Endophytes can be defined as microbial communities (especially bacteria and fungi) that are found inside plant tissues without causing any apparent harm to the host. The presence of seed-borne bacteria has been documented throughout seed maturing stages of rice^14^ and in the endosphere of mature rice seeds^15^. From a rice plant which was grown in the field, Bertani et al. isolated a total of 1,318 putative bacterial endophytes and made a working collection of 229 isolates^16^. In another study, a total of 4,155 bacterial operational taxonomic units (OTUs) and 1,679 fungal OTUs in samples of rice sprouts, stems, and roots were analyzed^17^. Verma et al. showed that seeds of rice naturally harbor bacterial endophytes that play key roles in modulation of seedling development^18^. Also, some endophytes were shown to have biocontrol ability and plant-growth-promoting functions^19^. However, the function and ecology of the great majority of bacterial endophytes inside the host plant is unknown.

In this study, multiple rice seed born bacteria (RSB) were identified which showed an ability to suppress the virulence of *B. glumae*. Furthermore, the distribution of endogenous bacteria in plants that had been treated with the pathogen and RSBs changed dramatically. The result indicated a possible novel pathogen control technology mediated through endogenous microbiota. This will be also a nice model to clarify the mechanism of multiplication of opportunistic pathogens such as *B. glumae*.

## Results

### Pregerminated seedlings are more tolerant to *B. glumae* infection

**Figure 1:**
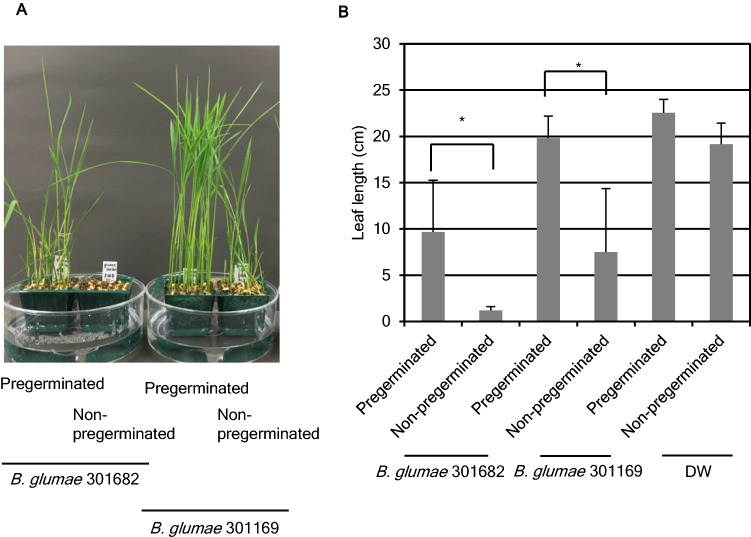
Pregerminated rice seeds were more tolerant to *B. glumae* infection than non-pregerminated ones. Pregerminated and non-pregerminated rice seeds were inoculated with the suspension of *B. glumae* 301169 and *B. glumae* 301682 under reduced pressure. (**A**) Photographs of the seedlings at 8 dpi. (**B**) The leaf length of the seedlings were measured at 8 dpi (n = 25). Statistical significance was subsequently determined through Student’s t-test, *p < 0.0001. Results are representative of four experiments.

The virulence of *B. glumae* on both pregerminated rice seeds and non-pregerminated seeds was tested. As shown in Fig. 1A,B, severe growth disruption was observed when non-pregerminated seeds were treated with *B. glumae* 301682. In contrast, the growth of pregerminated seeds treated with the pathogen was unaffected. A similar trend was shown in the case of *B. glumae* 301169, although it showed lower virulence than 301682 infection. A water-treated control showed normal growth. To quantify the difference in virulence, bacterial titer in the infected seedlings was analyzed (Fig. S1). Surprisingly, the number of bacteria in pregerminated seedlings was higher than in non-pregerminated seed from 1 through 4 days post inoculation (dpi). Furthermore, bacteria were also detected in the non-inoculated seed. This showed that the virulence would not simply be correlated to the population size of endogenous bacteria.

### Isolation and identification of the contaminated endophyte

**Figure 2:**
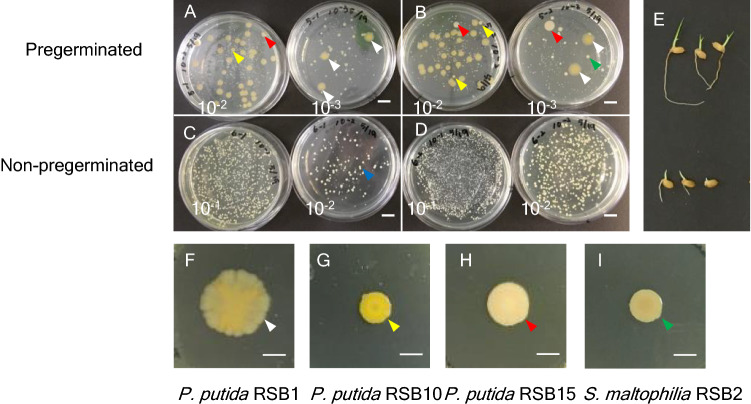
Diverse bacterial population in pregerminated seedlings and identified RSBs. Pregerminated and non-pregerminated rice seeds were inoculated with *B. glumae* 301682 (OD = 0.0004) under reduced pressure. (**A**–**D**) The bacterial population at 4 dpi. A lysate from whole seedlings was plated on an LB plate supplemented with Chloramphenicol at the indicated dilutions. Two independent sets (**A**,**B** for pregerminated, **C**,**D** for non-pregerminated) of the experiments are shown. Bars, 1 cm (**E**) Photographs of the 4 dpi seedlings. Top panel shows pregerminated, bottom panel shows non-pregerminated. (**F**–**I**) Colonies of rice seed-born (RSB) microbes isolated from pregerminated seedlings. Bars, 1 mm Photographs are taken after 5 d on NA medium. White arrow shows RSB1, yellow is RSB10, red is RSB15, green is RSB2 and blue is *B. glumae* on (**A**,**B**,**F**,**G**,**H**,**I**). Results are representative of two experiments.

The shape and color of bacteria grown from pregerminated seeds varied (Fig. 2A,B). In contrast, the bacteria from non-pregerminated seedlings were homogenous (Fig. 2C,D). Thus, it was speculated that the pregerminated seeds carried endophytic bacteria other than the inoculated pathogen. Supporting this, bacteria were also detected in seedlings treated with water only (Fig. S1). Furthermore, bacteria in the pregerminated seedlings were detected from 1 dpi, while bacteria from non-pregerminated seeds were detected only from 3 dpi. This strongly suggested that seedborne endophytic bacteria that multiplied during the pre-germination period, before pathogen treatment, control the pathogen growth in the plant.

Next, endophytic bacteria were isolated from each colony derived from endophytic tissue of 4 dpi plants (Fig. 2E). Three isolates of *P. putida* and one of *S. maltophilia* were identified by their 16S rRNA gene sequence (Figs. S2, S3) and Phylogenic tree analysis (Fig. S4). As shown in Fig. 2F–I, the three *P. putida* showed different hues and morphologies, and thus were named RSB1, RSB10, and RSB15. The *S. maltophilia* was named RSB2.

### Biocontrol effects of the isolated bacteria

**Figure 3:**
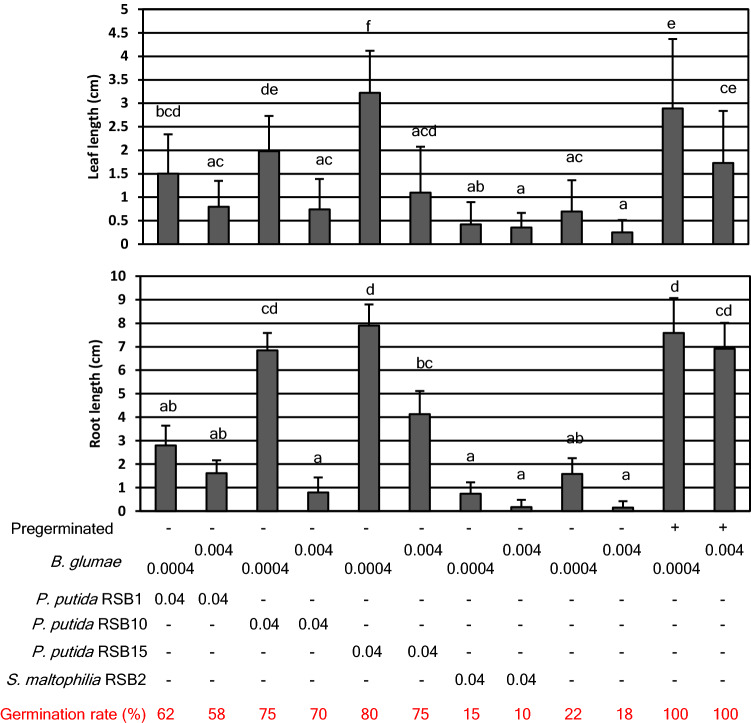
The growth inhibition caused by *B. glumae* was suppressed by *P. putida* RSB treatment. RSBs were inoculated on non-pregerminated rice seeds with or without *B. glumae* 301682, and leaf length (upper) and root length (bottom) was measured at 8 dpi (n = 25). Infection by *B. glumae* on pregerminated seeds is shown in the right two columns. Numbers under the figure show optical density of each bacteria in the inoculum. Statistical significance was subsequently determined through a TukeyHSD test (R software). Results are representative of three experiments.

**Figure 4:**
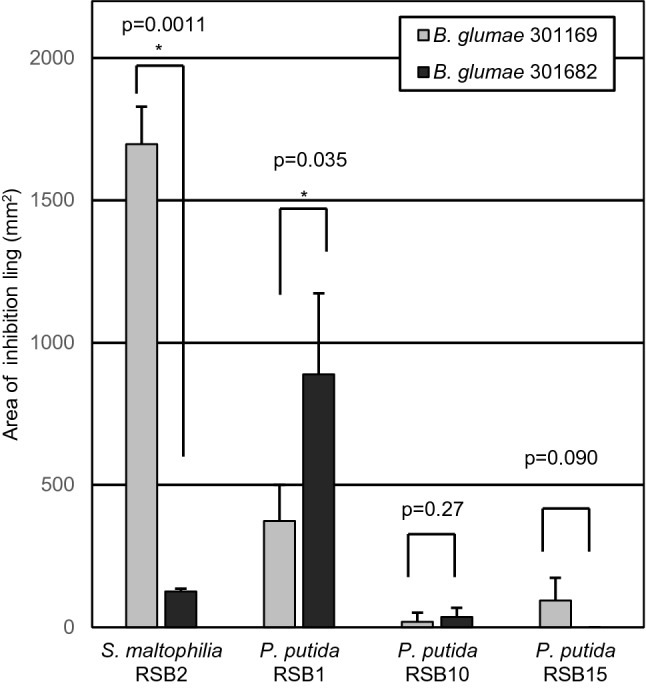
Competitive inhibition test of RSBs to *B. glumae* 301169 and 301682. RSBs were spotted on *B. glumae* 301169 or 301682-embedded plates. Area of inhibition ling (halo) was measured 7 days after spotting (n = 3). Statistical significance was subsequently determined through Student’s t-test, *p < 0.05. Results are representative of three experiments.

**Figure 5:**
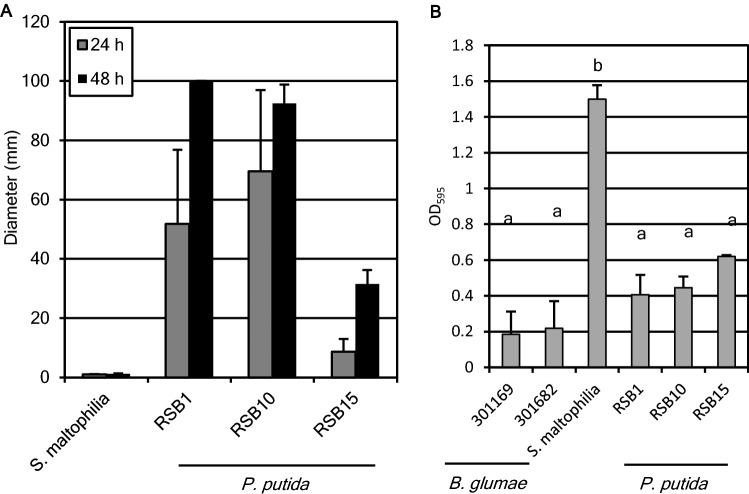
Characterization of RSBs. (**A**) Size of swarming (n = 5). (**B**) Biofilm formation assays of RSBs and *B. glumae* (n = 3). Statistical significance was subsequently determined through a TukeyHSD test (R software). Results are representative of two experiments.

To test the hypothesis that bacteria which multiplied during germination attenuated the virulence of *B. glumae,* isolated RSBs and *B. glumae* were co-inoculated on non-pregerminated seeds (Fig. 3). The virulence was indexed by measuring root and leaf length of infected seedlings. Each *P. putida* RSB treatment allowed root and leaf elongation in the presence of pathogen. In contrast, RSB2 did not protect against *B. glumae*. Thus, *P. putida* RSBs have the ability to suppress the pathogenicity caused by *B. glumae*. Next, a competitive inhibition assay was done with each RSB paired against different *B. glumae* strains. Strong inhibition zone (halo) was observed in the case of 301682 (base)–RSB1 (spot), 301169 (base)–RSB2 (spot), and mild halo was observed in the case of 301169 (base)–RSB1 (spot), as shown Fig. 4. This strongly suggested that RSB1 was competitive against the pathogen. Notably, RSB1 colonies multiplied to almost 20 mm diameter on the *B. glumae* 301169 plate, whereas size of the colonies on 301682 was under 10 mm diameter, even at 7 days after dropping bacterial suspension on the plate (Figs. S5, S6). This also indicated that RSB1 multiplied faster than RSB10 and RSB15. RSB2 was competitive against the weak pathogen but less so against the strong pathogen. A swarming motility test (Fig. 5A) revealed high mobility of *P. putida,* especially RSB1 and RSB10, while RSB2 had no mobility. Biofilm formation of *P. putida* RSBs was almost two times that of *B. glumae*, whereas RSB2 produced approximately 7 times more biofilm than *B. glumae* (Fig. 5B). The effects of RSBs on plant growth were tested (Fig. S7), demonstrating that RSB15 slightly but significantly promoted root growth.

### Cooperative effects of the endophyte

**Figure 6:**
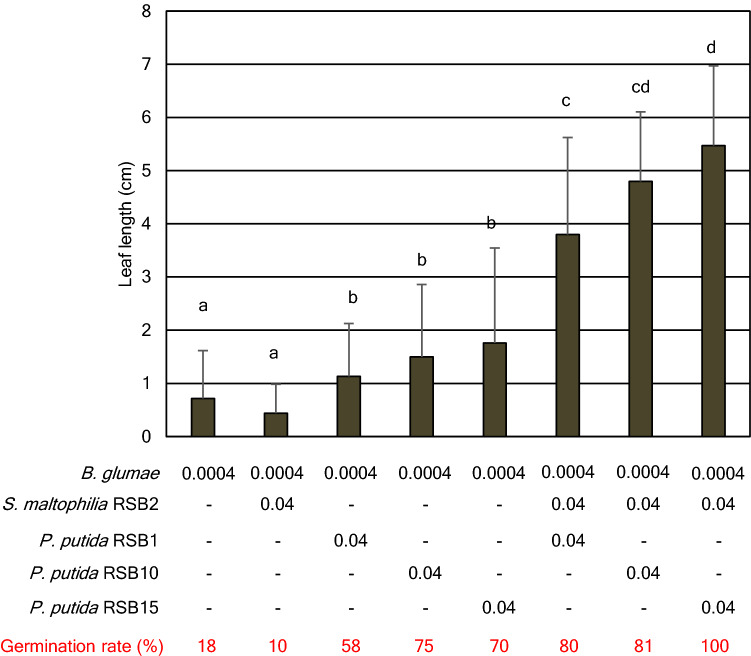
RSB1, RSB10, RSB15 and RSB2 coordinately suppressed growth inhibition caused by *B. glumae*. Leaf length of the seedlings were measured at 8 dpi (n = 20). Numbers under the figure indicate optical density of each bacteria in the inoculum. Statistical significance was subsequently determined through a TukeyHSD test (R software). Results are representative of four experiments.

Based on these contrasting characteristics of RSBs, it was speculated that both *P. putida* and *S. maltophilia* applications might strengthen the ability of *P. putida* to protect. Therefore, a combined application of each of the *P. putida* RSBs as well as the *S. maltophilia* RSB2 was tested. As shown in Fig. 6, Figs. S8, S9, the *P. putida* RSBs and RSB2 coordinately protect rice seedlings from *B. glumae* infection based on leaf length and disease index analysis. Notably, the combined application of RSB2 and RSB15 resulted in 100% germination (Fig. 6, Figs. S8, S9). To clarify the effects of high biofilm formation activity of RSB2 on the synergistic effects, biofilm formation mutants were screened. From approximately 1,000 UV random mutants, 20 mutants were selected by a first screen in microplates. Following a second screening in test tubes, two enhanced (#UV-17, 28) and two reduced (#UV-45, 60) biofilm formation mutants were selected (Fig. S10). The virulence of *B. glumae* supplemented with RSB2 and RSB15 mutants was tested (Fig. S11). It was revealed that one of enhanced biofilm formation mutants, #UV-17 showed the highest biofilm formation and slightly accelerated the protection effects. Furthermore, two of reduced biofilm mutants prevented the effects. The other enhanced mutant, #UV-28, did not show any significant change, and biofilm formation seemed insufficient to enhance protection. Overall, the biofilm formation activity of RSB2 was likely to be one the factors coordinating protection effects.

### RSBs application changed endogenous microbiota

**Figure 7:**
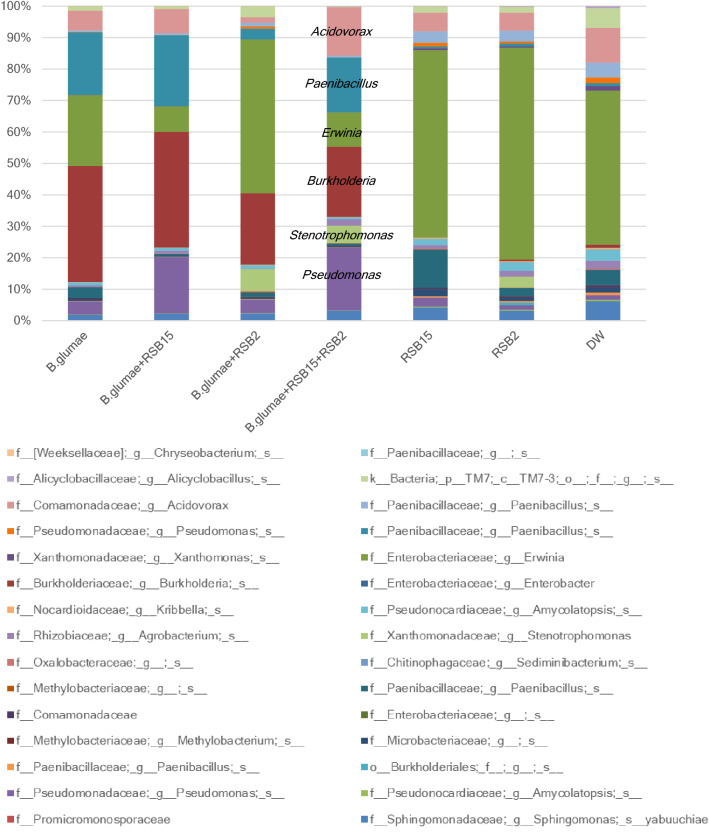
Percentage of OTUs detected in the samples collected from rice seedlings treated with *B. glumae* and RSB2 and RSB15. Four days after treating non-pregerminated seed with *B. glumae* (OD = 0.0004) and RSBs (OD = 0.04), twenty-five seedlings collected three times and mixed all of them and ground with liquid-nitrogen, and DNA was extracted for 3 sets (technical replications). Results from the analysis of 16S rRNA gene sequences detected by culture-independent evaluation of rice using next generation sequencing with a MiSeq system (n = 3). Box color pink, *Acidovorax*; blue, *Paenibacillus*; green, *Erwinia*; brown, *Burkholderia*; pale green, *Stenotrophomonas*; purple, *Pseudomonas*.

Application of RSBs resulted in reduced virulence of *B. glumae*. To understand how RSBs affected the microbial composition inside the seedlings, comprehensive microbial analysis was conducted using high throughput sequencing of 16S rRNA genes for bacteria, and the Internal Transcribed Spacer (ITS) of ribosomal arrays for fungi. As shown in Fig. 7, the bacterial genera *Burkholderia* and *Paenibacillus* increased in relative abundance following *B. glumae* treatment, while *Erwinia* decreased. In detail, the bacterial genera *Burkholderia* at Water (0.92%), RSB2 (0.40%), RSB5 (0.040%) was changed to *B. glumae* (37%), *B. glumae* and RSB15 (37%), *B. glumae* and RSB2 (23%), *B. glumae* and RSB2 and RSB15 (22%). The virulence of *B. glumae* was suppressed by RSB15 treatment, but the relative abundance of the *Burkholderia* OTU had not changed (*p* = 0.49; Students t-test). Additional treatment of RSB2 to *B. glumae* and RSB15 decreased the relative abundance of the *Burkholderia* OTU (*p* = 0.00003; Students t-test). The bacterial genus *Pseudomonas* (1.5% at water treatment) increased by additional treatment of RSB15 to *B. glumae* (18%, *p* = 0.00002; Students t-test), while the genus *Stenotrophomonas* (0% at water treatment) increased by additional treatment of RSB2 to *B. glumae* (7%, *p* = 0.0001; Students t-test). These results indicated that RSBs colonized and grew in the plant successfully. The bacterial genus *Acidovorax* (1.8% at *B. glumae* and RSB2) increased by additional treatment of RSB15 (15%, *p* = 0.00002; Students t-test). The bacterial genus *Paenibacillus* (3.4% at *B. glumae* and RSB2) increased by additional treatment of RSB15 (17%, *p* = 0.0003; Students t-test). Based on the results, it was speculated that the bacteria belonging to the genera *Acidovorax* and *Paenibacillus* were also able to influence *B. glumae* infection. Because of resource limitations, NGS analysis was conducted only on whole plants. To determine the distribution of endophytes and the pathogen in developing seedlings, culturable bacteria numbers of several parts of the seedling (leaf, seed and root) were counted over time distinguished based on the time each colony appeared and the colony morphology (Fig. S12). Putative *B. glumae* colonies which appeared at 66 h with yellow pigment were detected mainly in the leaf parts but also both in seeds and roots.

By ITS analysis, using exactly the same DNA that was used for 16S rRNA gene sequencing (Fig. S13), the drastic change seen in 16S rRNA gene abundances was not observed, largely because *Simplicillium* accounted for approximately 90% of the out relative abundance.

## Discussion

Seeds can act as vectors for transgenerational transmission of endophytes^20^. In this study, rice seed endophytes unexpectedly multiplied during the pregermination period (germination before pathogen inoculation), a normal period in rice cultivation. Interestingly, pregermination fortifies the rice plant against *B. glumae* infection. RSBs were eventually isolated as endogenous microbes that grew in the seed following artificial inoculation of the pathogen. These results strongly suggested that rice seed endophytes might control disease severity. In particular, the seed associated pathogen *B. glumae* seemed to be greatly influenced by endophytes. Furthermore, treatment with the endophytes greatly influenced other members of the endogenous microbiota. To my knowledge, there have been no reports that endophytes control *B. glumae* virulence.

Four potential biocontrol agents for the plant endophytic pathogen *B. glumae* were isolated and identified. Several questions remain. Are these bacteria common in rice plants? In which part of the rice seedlings are they distributed? Without comparing whole genome sequences, it is hard to determine to what extent these bacteria are similar to previously discovered *P. putida* and *S. maltophilia* detected in rice seeds or seedlings. *P. putida* was found in the rhizosphere of rice seedlings cultivated in neutral-pH soil in the Netherland, while it was found in shoots of rice seedlings cultivated in acidic soil^21^. In the same experiment, *S. maltophilia* was detected in rhizosphere, roots, and shoots of rice seedlings cultivated in both soil types^21^. In maturing rice seeds on a rice plant cultivated in a paddy field in Japan, only *S. maltophilia* was found at the seed surface at the early maturing stage^22^. In contrast, during analysis of endophytes of rice seedlings by the same group, using the same rice cultivar, neither *P. putida* nor *S. maltophilia* was detected^15^. These results indicated that flora of rice might be dependent on habitat, environment, and cultivar. The seed *Nipponbare* used in this study was collected at a paddy field in Tsukuba, Japan in 2014 and preserved in a refrigerator. Thus, remarkably, the endophytes may have been established in the seeds during the cultivation process and remained dormant for five years in the seeds! In this study, seedlings collected for experiments had not been surface sterilized, so RSBs possibly existed on the plant surface or rhizosphere. However, since it was demonstrated that the supplementation of RSBs suppressed the virulence (Fig. 3), it is highly likely those bacteria multiplied inside the plant. In fact, from the NGS analysis of the microbes in the seedlings, the genera *Pseudomonas* and *Stenotrophomonas* were detected abundantly from only the correspondingly RSB-treated seedlings (Fig. 7). Also, putative *B. glumae* were detected from the leaf by culture-dependent analysis (Fig. S12). This strongly suggested that the pathogen preferentially colonized the phyllosphere. Supporting this idea, *B. plantarii,* a bacteria related to *B. glumae*, accumulated in aerenchyma and the intercellular spaces^23^. Further understanding of intra-plant localization may elucidate how the pathogen gains virulence despite coexisting with the endophytes *in planta*. Unexpectedly, the relative abundance of the *Burkholderia* OTUs did not change due to RSB15 treatment (Fig. 7). Furthermore, the relative abundance of *Burkholderia* OTUs was lower in the case of *B. glumae* and RSB2 treatment, which showed intense growth disruption. This indicated that the bacterial titer might not corelate with the virulence. However, the occupancy of the *Burkholderia* OTU relative to other OTUs reduced from 37 to 22%. Therefore, the disease symptoms of *B. glumae* could be due to the balance of endophytes. In contrast to the NGS analysis, putative *B. glumae* was abundant in the case of culture-dependent analysis (Fig. S12). This inconsistent results due to the different time points of sample preparation that 4 dpi plant was used for NGS analysis while 10 dpi plant was used for culture-dependent analysis and also the limitation of NGS analysis that counting all cell included dead cell.

In previous reports, various strains of *P. putida* have been isolated and shown to have biocontrol activity against not only bacteria, but also fungi or nematodes^24–26^. Along with these *P. putida* strains that showed antimicrobial activity^27,28^, RSB1 and RSB2 isolated in this report also showed antibacterial activity against the pathogen (Fig. 4). It is known that *B. glumae* secrets a toxin called toxoflavin that can inhibit the growth of bacteria in the genera *Escherichia*, *Shigella*, *Micrococcus*, and *Bacillus*^29,30^. Growth of RSB1, RSB10 and RSB2 on a plate embedded with the high toxoflavin secreting strain *B. glumae* 301682 was inhibited compared to growth on a plate embedded with the lower toxoflavin-secreting strain *B. glumae* 301169 (Figs. S5, S6).

Some *P. putida* strains also showed resistance to broad-spectrum antibiotics^31^. *P. putida* and *S. maltophilia* collected from ranch soil rhizospheres showed organophosphate-degrading features^32^. Metallo-β-lactamase, responsible for resistance to antibiotics, is coded in the genome of *S. maltophilia*^32^. RSBs were resistant to kanamycin, ampicillin and chloramphenicol, and possibly others (data not shown). Therefore, perhaps those strains were resistant to the multiple antibiotics because of the antibiotic mechanisms such as RND efflux pumps. Interestingly, on the chloramphenicol plate, no colonies were found for any bacteria other than the four strains identified in the study. In contrast, a variety of microbes, and 10 times more bacteria, were found on chloramphenicol-unsupplemented plates (data not shown). This suggested that antibiotic resistant microbes were screened by the plate used for growth curves, and it fortuitously and coincidentally served as a biocontrol bacteria selection plate. *Acidovorax* and *Paenibacillus,* that were detected by NGS (Fig. 7) but not present on the chloramphenicol plate, possibly do not have chloramphenicol resistance, and therefore would be ignored in the culture-based experiment.

Each *P. putida* RSBs derived from infected rice seedlings showed different morphology and characteristics. The 16S rRNA gene sequence of RSB1 was distinct from that of RSB10 or RSB15 (Fig. S3) and also in phylogenetic analysis (Fig. S4). Interestingly, RSB1 showed highly-competitive activity against the more pathogenic strain, *B. glumae* 301682 (Fig. 4). Although the 16S rRNA gene sequences of RSB10 and RSB15 were highly similar (Fig. S3), motility was very different (Fig. 5). Motility has been shown to affect endophytic colonization and bacterial mobility within host plants^19^. However, since RSB15, which showed low motility, had protective effects against *B. glumae* infection, motility might be not required for the protection. RSB15 also promoted root elongation (Fig. S7), and thus it is plausible that the strain also secretes a plant hormone such as phytohormone indole-3-acetic acid (IAA).

To my knowledge, there are no reports showing cooperative effects of *P. putida* and *S. maltophilia* for the control of a plant pathogen. Interestingly, cooperative work was reported between *S. maltophilia* and *P. aeruginosa* during polymicrobial airway infections^33^. In that study, microscopic analysis of biofilms formed in vitro revealed that *S. maltophilia* formed well-integrated biofilms with *P. aeruginosa*. Because the low biofilm formation strain weakened the cooperative work with RSB15 (Fig. S11), a molecule that promotes biofilm formation might accelerate the cooperation. Since application of RSB2 alone with *B. glumae* showed no protective effects, biofilm formed by RSB2 at normal densities was clearly not sufficient. Through the production of biofilms, RSB2 strains could easily adhere to the surfaces and aid in RSB2′s transmission. For *S. maltophilia* in human settings, the biofilm can also promote pathogen antibiotic tolerance, rendering some of the therapeutic options ineffective and causing setbacks in the selection of an appropriate treatment. This is consistent with the hypothesis that acceleration of biofilm formation of RSB15 with the help of RSB2 might protect plants from toxoflavin secreted by *B. glumae*. Further studies visualizing RSBs on site within biofilms will clarify the point. Altering the order of application of RSBs, for example, by including pre-treatment with RSB2 prior to other RSBs or pathogen, might enhance effects and deserves further investigation. It was also reported that members of the *S. maltophilia* and *B. cepacia* species complexes are typically multidrug resistant and excellent biofilm producers^32^. These phenotypes are controlled by quorum sensing systems from the diffusible signal factor (DSF) family. Resolving how DSF affects the interaction of *B. glumae* and RSBs would be an important future direction. High biofilm formation could be essential for the synergistic role of RSB2, and could be useful to promote effects by other *P. putida* that have been described in other studies or that are commercially available microbial inocula.

Many strains of *S. maltophilia* were isolated from various plants such as potato^34^, cucumber^35^, wheat^36^, and poplar^37^, and some of them showed plant growth promoting and disease suppressive effects. Most of those strains were reported to produce the phytohormone IAA^34–37^. However, the RSB2 isolated in this study was not shown to have any plant growth promoting (Fig. S7) nor disease suppression effects (Fig. 3) in the experimental conditions tested. Other forms of inoculation, such as soil treatment^34^, might be valuable to test.

Great numbers of strains belonging to *P. putida* and *S. maltophilia* are highly versatile and adapted to diverse habitats such as soil, water, the plant rhizosphere and endosphere, and occasionally as opportunistic pathogens in immune compromised patients. Thus, it would be essential to evaluate the environmental and human health impact prior to adoption as biocontrol material in the field. Many *S. maltophilia* described that are responsible for a variety of infections in both humans and animals. By the comparative whole genome analysis of 375 unique *S. maltophilia* from various origins such as humans, other animals, and the environment, it was determined that bacteria originating from environment were distinct from those isolated from humans or other animals^38^. This indicates that RSB2 is unlikely to gain pathogenicity towards humans or other animals. Further genomic analysis of RSB2 will be needed to be confirm that it indeed belongs to the group of environmental strains that cannot infect humans and other animals.

Since RSBs multiply easily and rapidly in either liquid LB (data not shown) or NA solid medium (Fig. 2), those could likely be easily cultured on a commercial scale. Seed treatment before sowing might save cultivation costs compared to spraying bacteria on the plant or pretreating field soil. Furthermore, RSBs could also increase protection even on the most resistant rice variety which has been developed. Although the enhanced protection ability of RSBs (Fig. 6), those rice still showed disease phenotype (Figs. S8, S9). Nonetheless, further evaluation of the effective concentration or inoculation methods are necessary. In addition, improvement of diagnostic tools for detection at early stages of rice development, or even at the seed stage, would be helpful to diminish the incidence of *B. glumae*. Further molecular analysis of the interaction between the pathogen and endophytes, including RSBs, will lead to novel factors that can be useful to detect traces of *B. glumae* in seeds or in planta.

Current global climate change may cause an increase in new or previously negligible diseases. Enhancement of agricultural production to feed rapidly an increasing population worldwide, paired with the development of sustainable agriculture, is essential. Characterization and application of endophytes to control plant pathogens is an effective and sustainable technique. Development of novel biocontrol agents derived from rice plants may aid in achieving these goals. Moreover, the development of novel technology for appropriate disease control using endophytes tailored to the actual disease, plant species, and environment could reduce current agricultural overuse of pesticides or fertilizer.

## Materials and methods

### Plant material and pathogenic organism

Rice ‘Nipponbare’ seeds harvested in the test field of NARO in Tsukuba city Japan and kept at 4 °C for more than two years were used. The pathogens *B. glumae* MAFF301682 (*B. glumae* 301682) and *B. glumae* MAFF301169 (*B. glumae* 301169) were provided by Genetic Resource center, NARO, Tsukuba. Spontaneously grown bacteria on Luria–Bertani (LB) medium supplemented with chloramphenicol were isolated and used for further inoculation as chloramphenicol resistant strains.

### Bacterial inoculation to rice seed

*Burkholderia glumae* strains were grown at 30 °C on LB medium supplemented with 1% agar and 10 μg/ml chloramphenicol for 3 days at 28 ± 2 °C and collected with sterilized distilled water (SDW) for inoculation. Rice endophytic bacteria isolated in the experiment RSBs were grown at 30 °C on LB medium supplemented with 1% agar and 10 μg/ml chloramphenicol for 24 h at 28 ± 2 °C and collected with SDW for inoculation. Approximately 20–25 rice seeds sterilized by soaking in Antiformin (available chlorine 5%) (Wako, Tokyo, Japan) for 10 min. The seeds were then rinsed with SDW several times. For pregermination, the sterilized seeds were soaked in sterilized water at 30 °C for 2 days in the dark. The pre-germinated or sterilized non-germinated rice seeds were soaked in 10 ml suspension of bacteria(s) adjusted to the indicated OD_600_ and held under a vacuum for 1 min. Following agitation for 10 min under normal air pressure, the inoculated seeds were dried on paper and sown in sterilized soil (Bonsol No. 2, Sumitomo Kagaku Kougyo, Japan). The inoculated seeds were incubated in a growth chamber at 28 °C under a 14-h photo period and 24 °C under 10-h dark period with 100% humidity. Disease rate was evaluated by the length of the leaf shelf and root and disease index at the indicated time after bacterial inoculation.

### Bacterial growth experiment in rice plant

Three whole plant samples were collected and washed in SDW three times and smashed by micro-smash (Bio-rad) with two stainless steel beads (4.8 mm) in a 2 ml sample tube with 1 ml SDW. Serial dilutions were prepared from the smashed suspension, and 100 μl aliquots from each dilution was spread on chloramphenicol-containing medium and incubated for 3 days at 28 ± 2 °C. Morphologically distinct bacterial colonies were each picked and spread on a new LB medium for further purification. The purified isolates were preserved in 20% glycerol solution at − 80 °C.

### Molecular characterization of bacterial isolates

Bacterial DNA was obtained using the DNeasy Blood &Tissue Kit (QIAGEN). PCR for 16S rRNA gene amplification was performed by using the bacterial-specific primers, 27F (5′-AAGGAGGGGATCCAGCCGCA-3′) and 1492R (5′- GTGCCAGCAGCCGCGG -3′). PCR amplifications were performed with KOD plus polymerase as described before^39^. The PCR product was purified using Wizard PCR Preps DNA Purification System (Promega, Madison, WI, USA). Purified double-stranded PCR fragments were directly sequenced with Big Dye Terminator Cycle sequencing kits (Applied Biosystems, Forster City, CA, USA).

### Biofilm formation

Biofilm formation was measured using the method previously reported^40^. Briefly, an overnight culture of bacteria (OD_600_ = 0.4) was diluted 1:100 in fresh sterile LB broth and 3 ml aliquots were removed to 13 ml sterilized tube (polypropylene) and incubated without agitation for 48 h at 28 °C. Planktonic bacteria were discarded, and the biofilms, which formed on the tube wall, were washed three times with distilled water. Into each tube, 4 ml of 0.1% crystal violet (FUJIFILM Wako Pure Chemical Co., Osaka, Japan) were added, and the tubes were incubated for 15 min at 30 °C. Excess crystal violet was then discarded, and stained biofilms were washed three times with 6 ml of distilled water. Finally, 70% ethanol was added to the stained biofilms, and the OD at 590 nm was read to assess the strength of biofilm formation. For small-scale biofilm mutant screening, 10 μl overnight culture of each independent bacteria was transferred to fresh 90 μl LB media in 96-well microtiter plates and incubated without agitation for 48 h at 30 °C. Biofilm cells were stained with 0.1% crystal violet and washed, the stain remaining in the cells was solubilized with 70% ethanol, and the optical density at 590 nm was determined.

### Swarming motility

Bacteria swarming was done following a previous report^41^ with some modifications. Briefly, 1 ml of an overnight culture of each strain was adjusted to OD_600_ 3.0, centrifuged and washed twice with SDW, and suspended in 100 μl SDW. To inoculate the plates, 5 μl bacteria suspension was spotted in the center. Plates consisted of modified M9 medium^41^, solidified with 0.5% Bacto-agar (Difco). Five replicates were performed for every test and the experiments.

### Competitive inhibition test

One ml of an overnight culture of each strain was adjusted to OD_600_ 3.0, centrifuged and washed twice with SDW, and suspended in 100 μl SDW. For making a bacterial embedded plates, 800 μl of a “base” bacterial suspension was added to 20 ml of LB agar pre-cooled to 42 °C, gently mixed, and then poured into one square petri dish. Two μl of each “spot” suspensions were spotted on “base” bacteria embedded plates. Area of inhibition ling (halo) and colony size was measured 7 days after dropping.

### Generation of mutants by UV-mutagenesis

Five ml of overnight culture of RSB2 was collected and resuspended into SDW. The bacterial suspension was diluted to 10^6^ CFU/ml and plated onto LB plates. After 90 min at 30 °C of preculture, UV (Germicidal f15t8/UVB) was irradiated in 1 min in the hood. Each colony that had emerged on the plate after 2 days at 30 °C was picked as a mutant candidate. The number of colonies on the UV irradiated plate was half of that of the non-irradiated plate. Around 1,000 mutant candidates were applied to the first step screening using the mini-scale biofilm formation test described above in a microtiter plate. By the first step of screening, 20 mutants were picked as biofilm formation mutants. Among these candidates, 2 of the mutants that enhanced biofilm formation and 2 of the mutants that suppressed biofilm formation selected for further experiments.

### NGS analysis

To assess the structure of the microbial community in the rice seedlings, 16S rDNA gene and ITS amplicon sequencing using MiSeq was performed. Four days after treating non-pregerminated seed with *B. glumae* (OD = 0.0004) and RSBs (OD = 0.04), twenty-five seedlings collected three times and mixed all of them and ground with liquid-nitrogen, and DNA was extracted for 3 sets (technical replications) of each ground sample using the DNeasy Plant Mini Kit (Qiagen, USA) according to the manufacturer’s instructions. The extracted DNA was adjusted with distilled water to a concentration of 30 ng/μl in a total volume of 50 μl. The DNA concentration was fluorometrically determined using Qubit Assay Kits (Thermo Fisher Scientific Inc., Waltham, MA, USA) and a Nanophotometer (Implen GmbH, Munich, Germany). Sequence analysis of the rice samples using a MiSeq system (Illumina, Inc., San Diego, CA, USA) was performed by Fasmac Co., Ltd (Atsugi, Japan) as described before^42^. In brief, the forward primer 1st_PCR_515F (5′-ACA CTC TTT CCC TAC ACG ACG CTC TTC CGA TCT—[GTG CCA GCM GCC GCG GTA A]-3′) and the reverse primer 1st_PCR_806R (5′-GTG ACT GGA GTT CAG ACG TGT GCT CTT CCG ATC T—[GGA CTA CHV GGG TWT CTA AT]-3′). The second PCR reaction included two μl of the purified template DNA, 10 µM of the forward primer 2nd_F (5′-[AAT GAT ACG GCG ACC ACC GAG ATC TAC AC]—[XXXXXXXX]—[ACA CTC TTT CCC TAC ACG ACG C]-3′) and the reverse primer 2nd_R (5′-[CAA GCA GAA GAC GGC ATA CGA GAT]—[YYYYYYYY]—[GTG ACT GGA GTT CAG ACG TGT G]-3′). The PCR amplicons were mixed and subjected to 2 × 250 bp paired-end sequencing using MiSeq System v2. Cluster formation was performed using a MiSeq Reagent Kit v2 and PhiX Control Kit v3, and sequence analysis was performed using MiSeq Control Software ver 2.4.1.3. Real Time Analysis ver 1.18.54 and bcl2fastq ver 1.8.4. Analysis of the sequencing results included trimming of the primer region using Fastx toolkit, joining of the forward and reverse reads using FLASH, and quality filtering with sickle tool. The 97% identity OTU clustering and chimera filtering were performed using UCHIME (USEARCH package v8.0.1623) in QIIME, version 1.9.0. These data were then used to assign taxonomy against the Greengenes 13_8 database with a 97% similarity threshold using the UCLUST v1.2.22q in the assign taxonomy script of QIIME. Raw sequence counts were normalized by converting them to relative abundances through total sum scaling; for each sample, the sequence counts for each taxa was divided by the total number of sequence counts for that sample, and the result was converted to a percentage.

### DNA deposition

Culture-independent MiSeq sequence reads of the 16S rRNA gene and ITS are available in the DDBJ sequence read archive (DRA): PRJDB10214.

### Statistical analysis

Statistical analyses were carried out by use of R software version 3.6.1^43^. Tukey’s HSD test was done using library (multcomp^44^) and command cld to show significance.

## Supplementary Information

Supplementary Figures.
