# Supplementary material for: Multiple endogenous seed-born bacteria recovered rice growth disruption caused by Burkholderia glumae
Source: Sci Rep. 2021 Feb 18;11:4177. doi: 10.1038/s41598-021-83794-w (PMC7892555; doi:10.1038/s41598-021-83794-w)
Supplement: Supplementary file 1 — Supplementary Figures. [file 41598_2021_83794_MOESM1_ESM.pdf]

Multiple endogenous seed-born bacteria recovered rice growth disruption caused by *Burkholderia glumae*

Chiharu Akimoto-Tomiyama*
*Corresponding author: Chiharu Akimoto-Tomiyama at Plant and Microbial Research Unit, Division of Plant and Microbial Sciences, Institute of Agrobiological Sciences, National Agriculture and Food Research Organization, Tsukuba, Ibaraki 305-8518, Japan
akimotoc@affrc.go.jp

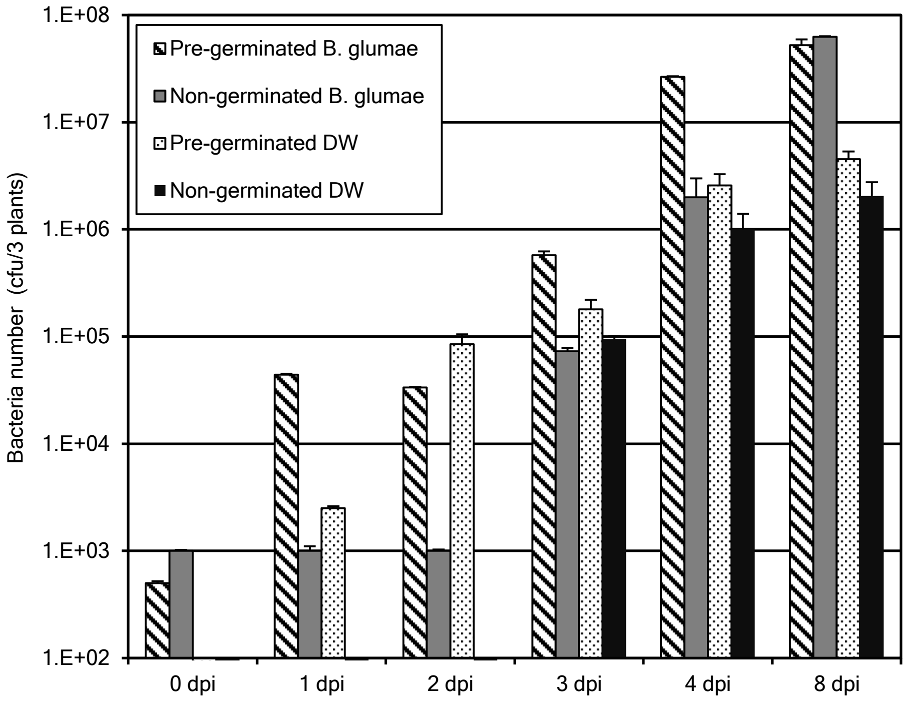

fig. S1 Numbers of endogenous bacteria in pregerminated and non-pregerminated seedlings with or without *B. glumae* inoculation (n=3). Results are representative of three experiments.

RSB1
CATAAGCGAGCGATGAGTAATTAATTAAGTTGAATGTGACAAGCCCCCGGACTCAAACGTGGGAACCTGCATCCAAACTGGCAAAGCTA
GAGTACGGTAGAGGGTGGTGGAATTTCCCGGGTAAACGGTGAAATAAGTAGAAATAGAAAGGAACACCAGTGGCGAAGGCGACCCAC
CTGGACTGATACTGACACTGAGGTGCGAAAGCGTGGGAAGCAAACAGGATTAGATACCTGGTAGTCCACGCCGTAAACGATGTCAAAC
TAGCCGTTGGAAATCCTTGAGATTTTAGTGGCGCAGCTAACGCATTAAGTTGACCGCCTGGGGAGTACGGCCGCAAGGTTAAAACTCAA
ATGAATTGACGGGGGCCCGCACAAGCGGTGGAGCATGTGGTTTAATTCGAAGCAACGCGAAGAACCTTACCAGGCCTTGACATGCAGA
GAACTTTCCAGAGATGGATTGGTGCCTTCGGGAACTCTGACACAGGTGCTGCATGGCTGTCGTCAGCTCGTGTCGTGAGATGTTGGGTT
AAGTCCCGTAACGAGCGCAACCCTTGTCCTTAGTTACCAGCACGTTATGGTGGGCACTCTAAGGAGACTGCCGGTGACAAACCGGAGGA
AGGTGGGGATGACGTCAAGTCATCATGGCCCTTACGGCCTGGGCTACACACGTGCTACAATGGTCGGTACAGAGGGTTGCCAAGCCGC
GAGGTGGAGCTAATCTCACAAAACCGATCGTAGTCCGGATCGCAGTCTGCAACTCGACTGCGTGAAGTCGGAATCGCTAGTAATCGCGA
ATCAGAATGTCGCGGTGAATACGTTCCCGGGCCTTGTACACACCGCCCGTCACACCATGGGAGTGGGTTGCACCAGAAGTAGCTAGTCT
AACCTTCGGGGGGACGGTTACCACGGTGTGATTCATGACTGGGGTGAAGTCGAACAAAGAAGCTAAGACCGCCCCT

RSB10
ACGAGGGCTATGGCCTCTTTGTTTCGACTTCACCCCAGTCATGAATCACACCGTGGTAACCGTCCCCCCGAAGGTTAGACTAGCTACTTCT
GGTGCAACCCACTCCCATGGTGTGACGGGCGGTGTGTACAAGGCCCGGGAACGTATTCACCGCGACATTCTGATTCGCGATTACTAGCG
ATTCCGACTTCACGCAGTCGAGTTGCAGACTGCGATCCGGACTACGATCGGTTTTGTGAGATTAGCTCCACCTCGCGGCTTGGCAACCCT
CTGTACCGACCATTGTAGCACGTGTGTAGCCCAGGCCGTAAGGGCCATGATGACTTGACGTCATCCCCACCTTCCTCCGGTTTGTCACCG
GCAGTCTCCTTAGAGTGCCCACCATAACGTGCTGGTAACTAAGGACAAGGGTTGCGCTCGTTACGGGACTTAACCCAACATCTCACGAC
ACGAGCTGACGACAGCCATGCAGCACCTGTGTCAGAGTTCCCGAAGGCACCAATCCATCTCTGGAAAGTTCTCTGCATGTCAAGGCCTG
GTAAGGTTCTTCGCGTTGCTTCGAATTAAACCACATGCTCCACCGCTTGTGCGGGCCCCCGTCAATTCATTTGAGTTTTACCTTGCGGCCG
TACTCCCCAGGCGGTCAACTTAATGCGTTAGCTGCGCCACTAAAATCTCAAGGATTCCAACGGCTAGTTGACATCGTTTACGGCGTGGAC
TACCAGGGTATCTAATCCTGTTTGCTCCCCACGCTTTCGCACCTCAGTGTCAGTATCAGTCCAGGTGGTCGCCTTCGCCACTGGTGTTCCTT
CCTATATCTACGCATTTCACCGCTACACAGGAAATTCCACCACCCTCTACCGTACTCTAGCTTGCCAGTTTTGGATGCAGTTCCCAGGTTG
AGCCCGGGGCTTTCACATCCAACTTAACAAACCACCTACGCGCGCTTTACGCCCAGTAATTCCGATTAACGCTTGCACCCTCTGTATTACC
GCGGCTGCTGGCACAAGTTAA

RSB15
GACATGCTCCTTGTTCGACTTCCCCCAGTCATGAATCACACCGTGGTAACCGTCCTCCCGAAGGTTAGACTAGCTACTTCTGGTGCAACCC
ACTCCCATGGTGTGACGGGCGGTGTGTACAAGGCCCGGGAACGTATTCACCGCGACATTCTGATTCGCGATTACTAGCGATTCCGACTTC
ACGCAGTCGAGTTGCAGACTGCGATCCGGACTACGATCGGTTTTGTGAGATTAGCTCCACCTCGCGGCTTGGCAACCCTCTGTACCGACC
ATTGTAGCACGTGTGTAGCCCAGGCCGTAAGGGCCATGATGACTTGACGTCATCCCCACCTTCCTCCGGTTTGTCACCGGCAGTCTCCTTA
GAGTGCCCACCATAACGTGCTGGTAACTAAGGACAAGGGTTGCGCTCGTTACGGGACTTAACCCAACATCTCACGACACGAGCTGACGA
CAGCCATGCAGCACCTGTGTCAGAGTTCCCGAAGGCACCAATCCATCTCTGGAAAGTTCTCTGCATGTCAAGGCCTGGTAAGGTTCTTCG
CGTTGCTTCGAATTAAACCACATGCTCCACCGCTTGTGCGGGCCCCCGTCAATTCATTTGAGTTTTACCTTGCGGCCGTACTCCCCAGGCG
GTCAACTTAATGCGTTAGCTGCGCCACTAAAATCTCAAGGATTCCAACGGCTAGTTGACATCGTTTACGGCGTGGACTACCAGGGTATCT
AATCCTGTTTGCTCCCCACGCTTTCGCACCTCAGTGTCAGTATCAGTCCAGGTGGTCGCCTTCGCCACTGGTGTTCCTTCCTATATCTACGC
ATTTCACCGCTACACAGGAAATTCCACCACCCTCTACCGTACTCTAGCTTGCCAGTTTTGGATGCAGTTCCCAGGTTGAGCCCGGGGCTTT
CACATCCAACTTAACAAACCACCTACGCGCGCTTTACGCCCAGTAATTCCGATTAACGCTTGCACCCTCTGTATTACCGCGGCTGCTGGCA
CA

RSB2
TGGCGGGAGGCCTAACCATGCAAGTCGAACGGCAGCCACAGTAAGAGCTTGCTCTTATGGGTGGCGAGTGGCGGACGGGTGAGGAAT
ACATCGGAATCTACTTTTTCGTGGGGGATAACGTAGGGAAACTTACGCTAATACCGCATACGACCTACGGGTGAAAGCAGGGGACCTTC
GGGCCTTGCGCGATTGAATGAGCCGATGTCGGATTATCTAGTTGGCGGGGTAAAGGTCCACCAAGGCGACGATCCGTAGCTGGTCTGA
TAGGATGATCAGCCACACTGGAACTGAGACACGGTCCAGACTCCTACGGGAGGCAGCAGTGGGGAATATTGGACAATGGGCGCAAGC
CTGATCCAGCCATACCGCGTGGGTGAAGAAGGCCTTCGGGTTGTAAAGCCCTTTTGTTGGGAAAGAAATCCAGCCGGCTAATACCTGGT
TGGGATGACGGTACCCAAAGAATAAGCACCGGCTAACTTCGTGCCAGCAGCCGCGGTAATACGAAGGGTGCAAGCGTTACTCGGAATT
ACTGGGCGTAAAGCGTGCGTAGGTGGTCGTTTAAGTCCGTTGTGAAAGCCCTGGGCTCAACCTGGGAACTGCAGTGGATACTGGGCGA
CTAGAGTGTGGTAGAGGGTAGCGGAATTCCTGGTGTAGCAGTGAAATGCGTAGAGATCAGGAGGAACATCCATGGCGAAGGCAGCTA
CCTGGACCAACACTGACACTGAGGCACGAAAGCGTGGGGAGCAAACAGGATTAGATACCCTGGTAGTCCACGCCCTAAACGATGCGAA
CTGGATGTTGGGTGCAATTTGGCACGCAGTATCGAAGCTAACGCGTTAAGTTCGCCGCCTGGGGAGTACGGTCGCAAGACTGAAACTCA
AAGGAATTGACGGGGGCCCGCACAAGCGGTGGAGTATGTGGTTTAATTCGATGCAACGGGAAGGAACATTACATGACCAAAACATGTC
GAGAACTTTCCAGAGATGGATGGGTACCTTAGGGAACTCGAACACAGGTGCTGCATGGCTGTCGTCAGCTCGTGTCGTGAGATGTTGA
GTTAAGTCCCGCAACGAGCGCAACCCTTGTCCTTAGTTGCCAGCACGTAATGGTGGGAACTCTAAGGAGACCGCCGGTGACAAACCGGA
GGAAGGTGGGGATGACGTCAAGTCATCATGGCCCTTACGGCCAGGGCTACACACGTACTACAATGGTAGGGACAGAGGGCTGCAAGCC
GGCGACGGTAAGCCAATCCCAGAAACCCTATCTCAGTCCGGATTGGAGTCTGCAACTCGACTCCATGAAGTCGGAATCGCTAGTAATCG
CAGATCAGCATTGCTGCGGTGAATACGTTCCCGGGCCTTGTACACACCGCCCGTCACACCATGGGAGTTTGTTGCACCAGAAGCAGGTA
GCTTAACCTTCGGGAGGGCGCTTGCCACGGTGTGGCCCGATGACTGGGGTGAAGTCCGAAACAA

fig. S2 16S rRNA gene sequences of RSBs.

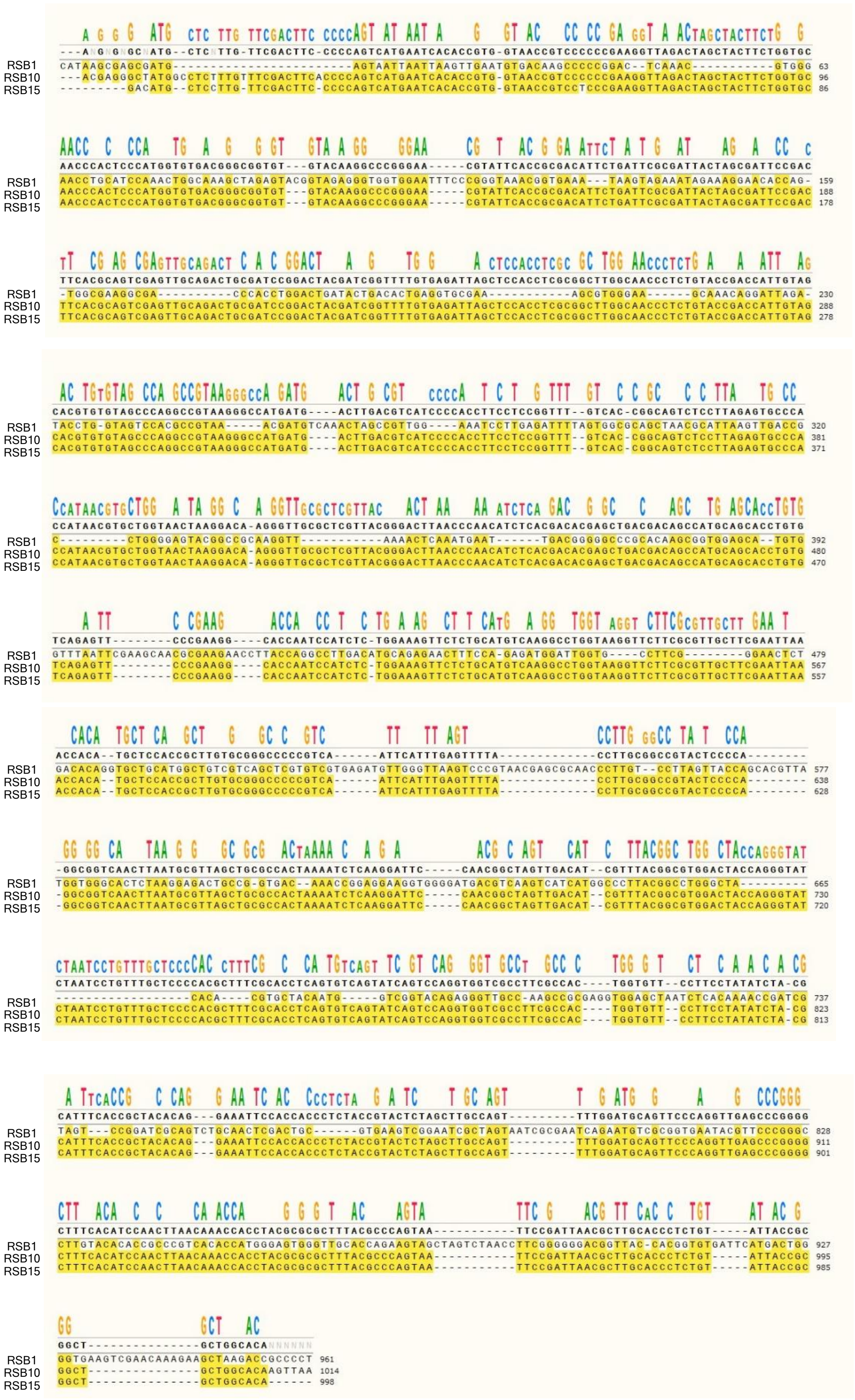

fig. S3 Alignment of *P. putida* RSB 16S rDNA gene sequences. The alignment tool in Snapgene was used for the analysis.

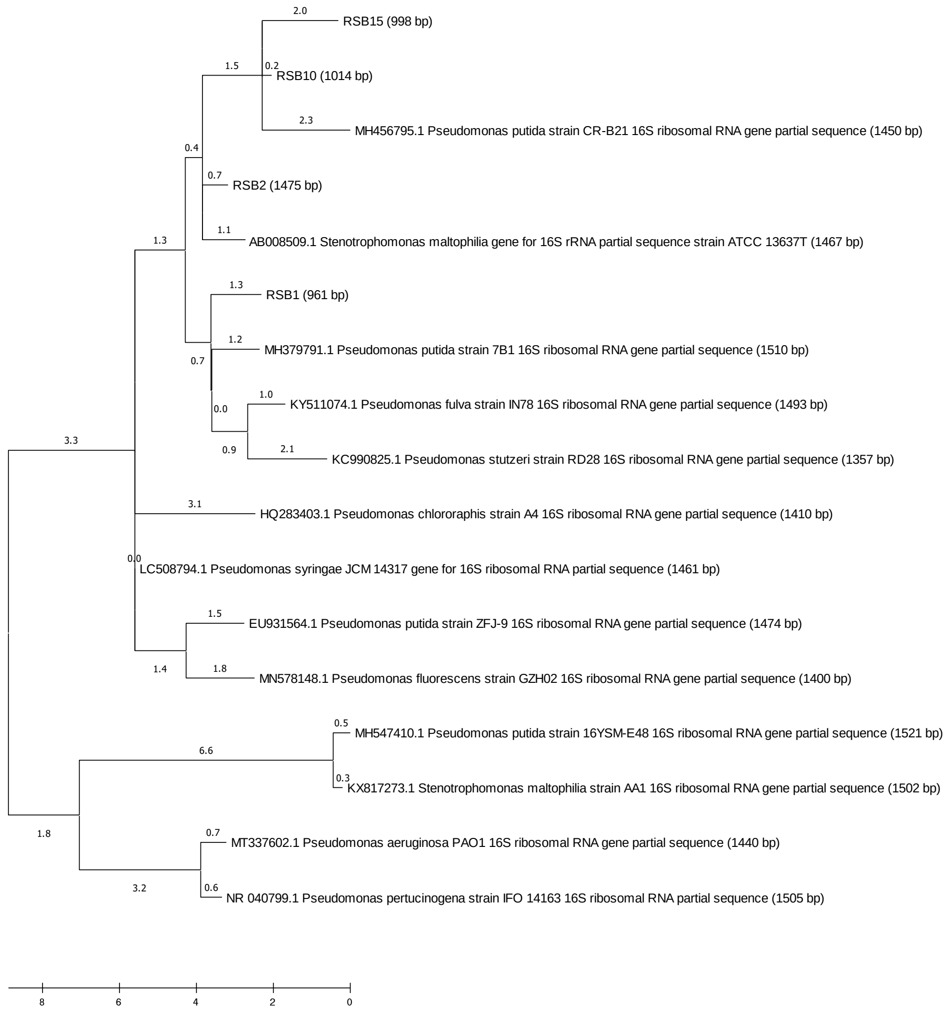

fig. S4 Sequence of RSBs 16S rRNA was analyzed with representative *Pseudomonas* 16S rRNA by Maximum Likelihood method.

The evolutionary history was inferred by using the Maximum Likelihood method and Tamura-Nei model [1]. The tree with the highest log likelihood (-31848.30) is shown. Initial tree(s) for the heuristic search were obtained automatically by applying Neighbor-Join and BioNJ algorithms to a matrix of pairwise distances estimated using the Tamura-Nei model, and then selecting the topology with superior log likelihood value. This analysis involved 17 nucleotide sequences. There were a total of 1521 positions in the final dataset. Evolutionary analyses were conducted in MEGA X [2].

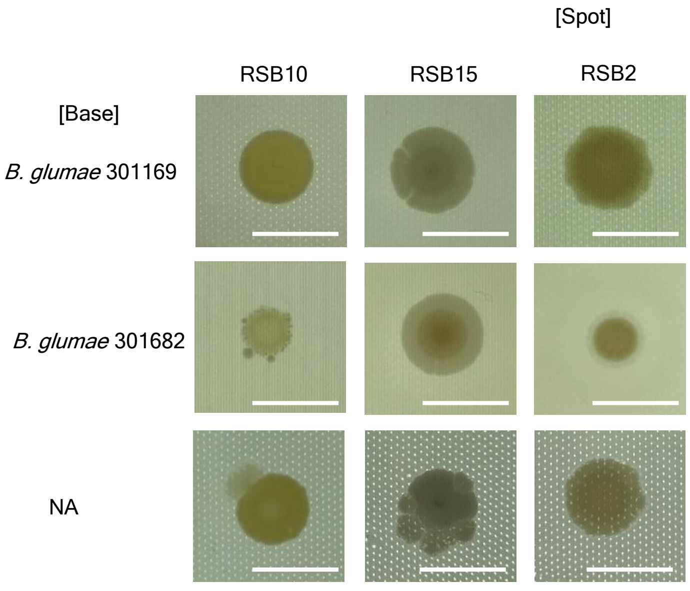

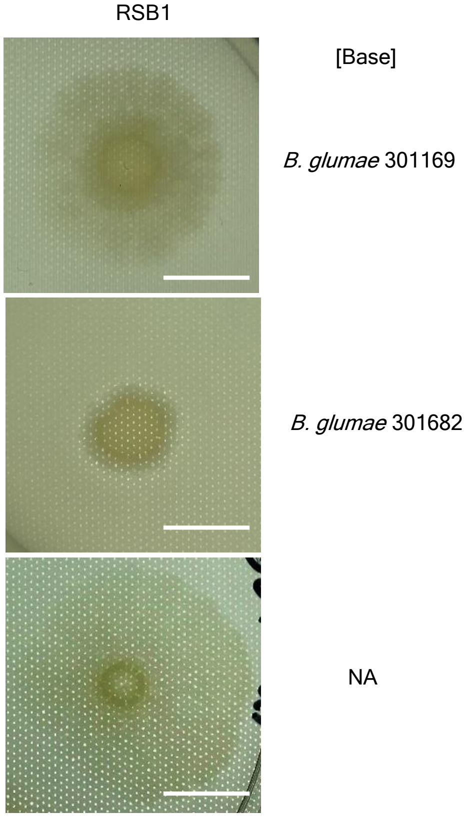

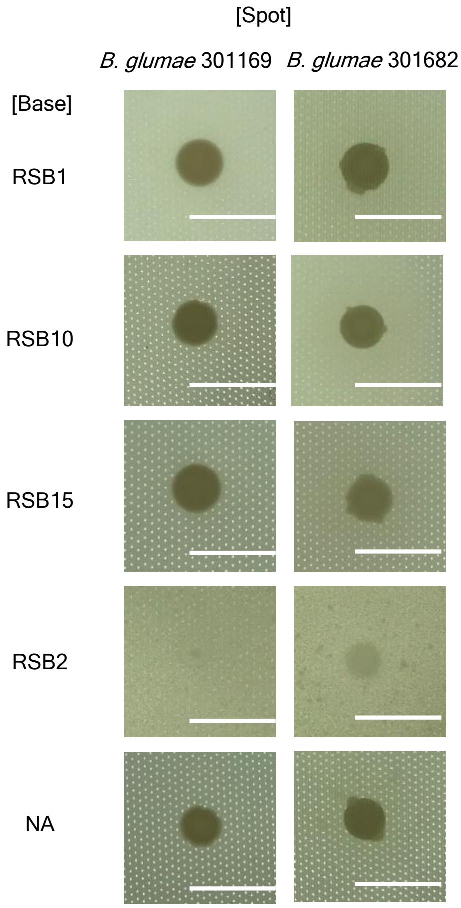

fig. S5 Pictures of competitive inhibition test of RSBs vs. *B. glumae* 301169 and 301682.
Bacteria were spotted on the embedded plate. Pictures were taken at 7 days after spotting. Results are representative of three experiments. Bars=1cm

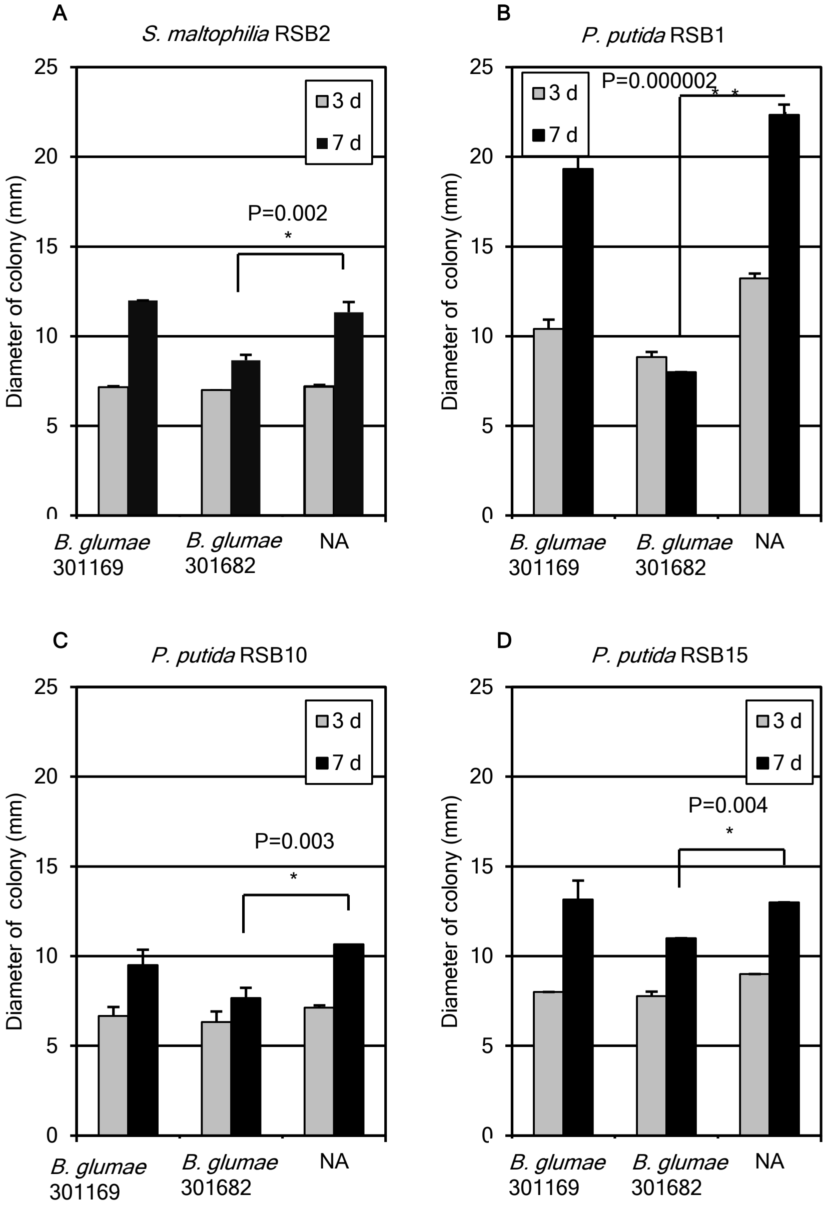

fig. S6 Colony size in competitive inhibition test of RSBs to *B. glumae* 301169 and 301682.

RSBs were spotted on *B. glumae* 301169 or 301682-embedded plates. Colony size was measured 3 and 7 days after spotting (n=12). NA indicates non-pathogen-embedded NA plate. A, *S. maltophilia* RSB2; B, *P. putida* RSB1; C, *P. putida* RSB10; D, *P. putida* RSB15. Statistical significance was subsequently determined through Students t-test., *p<0.01, * * p<0.0001. Results are representative of three experiments.

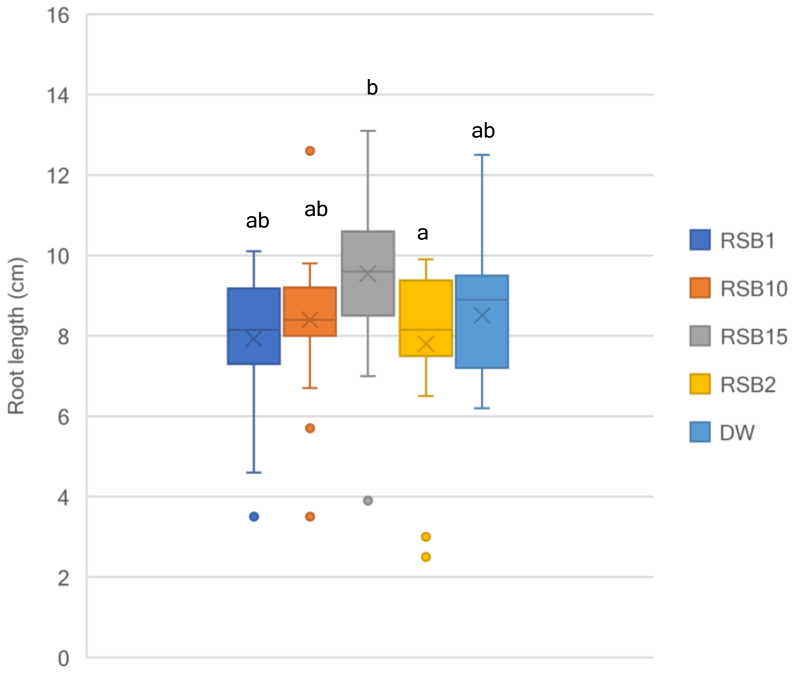

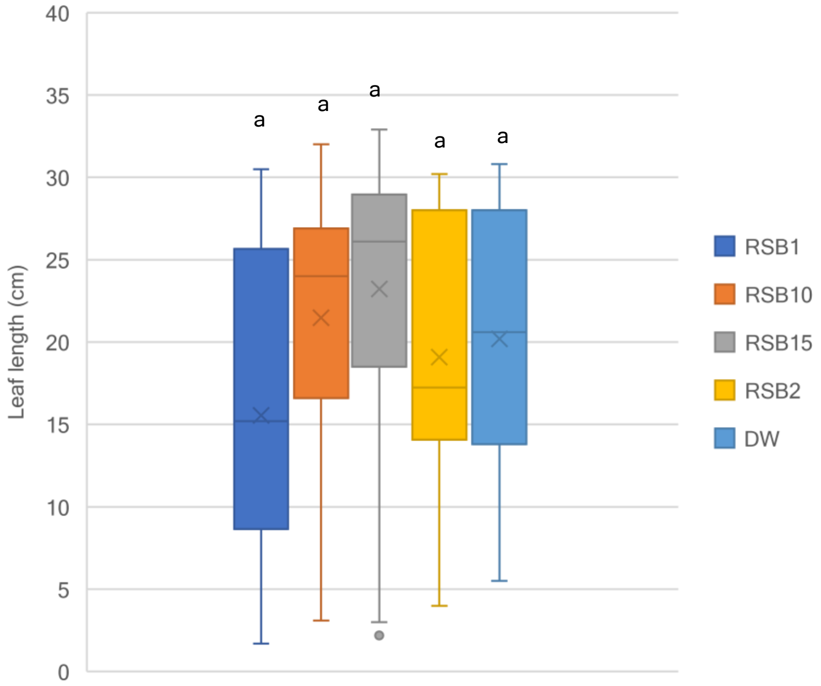

fig. S7 The growth promotion effects by RSBs.
The root (upper) and leaf (lower) length of rice seedlings 12 days after RSBs treatment (n=25). Statistical significance was subsequently determined through a TukeyHSD test (R software). Results are representative of three experiments.

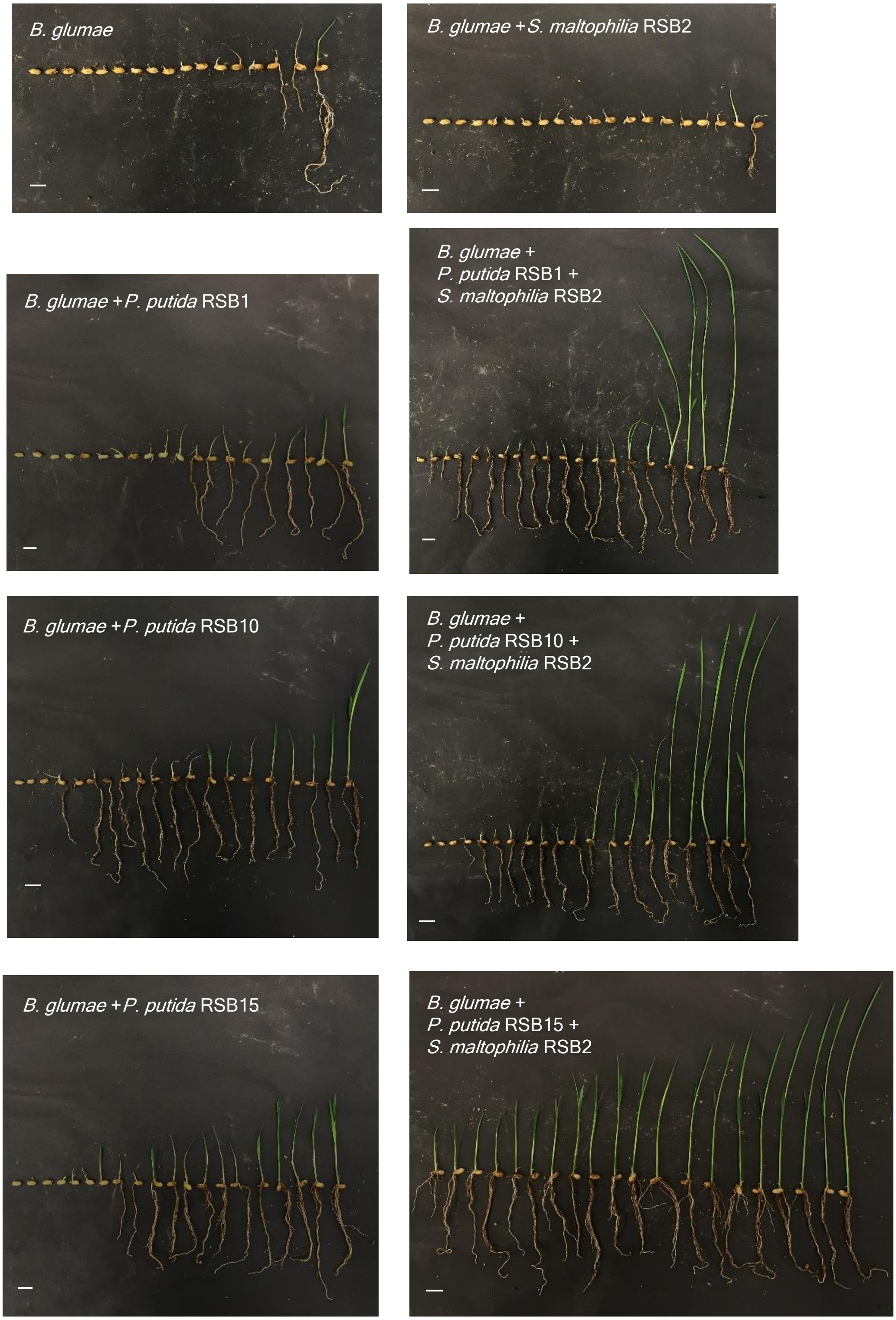

fig. S8 Pictures of rice seedlings treated with *B. glumae* and RSBs at 8 dpi.
B. glumae (OD=0.0004) and RSBs (OD=0.04) were treated to non-pregerminated seed. Results are representative of five experiments. Bars=1cm

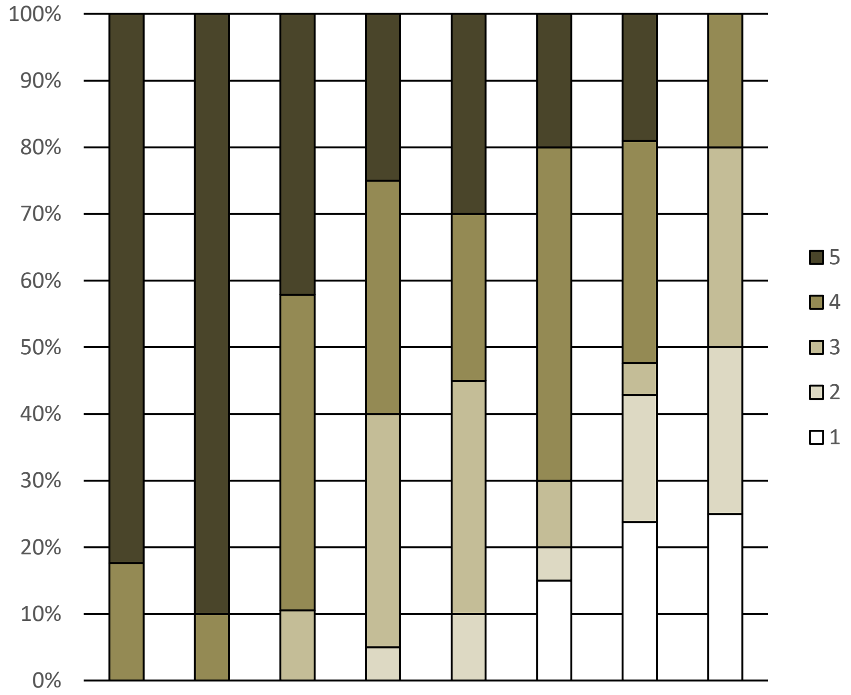

B. glumae	0.0004	0.0004	0.0004	0.0004	0.0004	0.0004	0.0004	0.0004
S. maltophilia RSB2	-	0.04	-	-	-	0.04	0.04	0.04
P. putida RSB1	-	-	0.04	-	-	0.04	-	-
P. putida RSB10	-	-	-	0.04	-	-	0.04	-
P. putida RSB15	-	-	-	-	0.04	-	-	0.04

fig. S9 Percentage of the disease index of the rice seedling treated with *B. glumae* and RSBs at 8 dpi.

B. glumae (OD=0.0004) and RSBs (OD=0.04) were treated to non-pregerminated seed (n=20~25). Disease index (DI) 5, no germination nor rooting; DI4, slight germination but no rooting; DI3, slight germination and rooting; DI2, leaf curving and blighting; DI1; partial leaf blighting. Results are representative of five experiments.

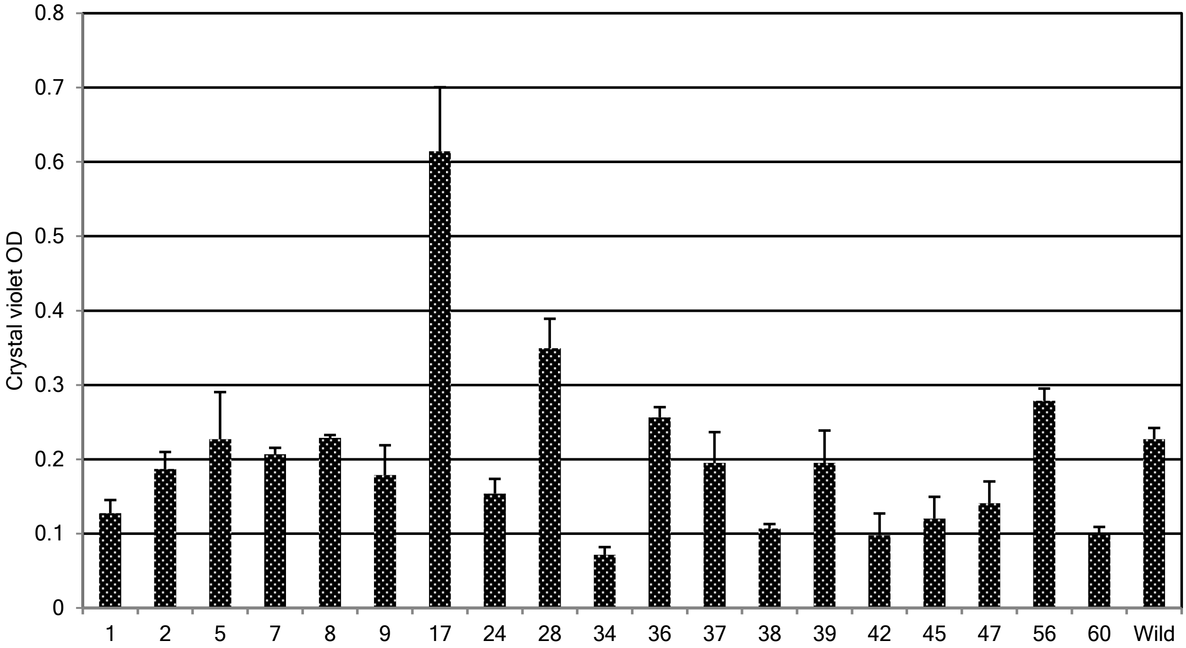

fig. S10 Biofilm formation test using UV-mutated RSB2 at second screening (n=3).
Results are representative of two experiments.

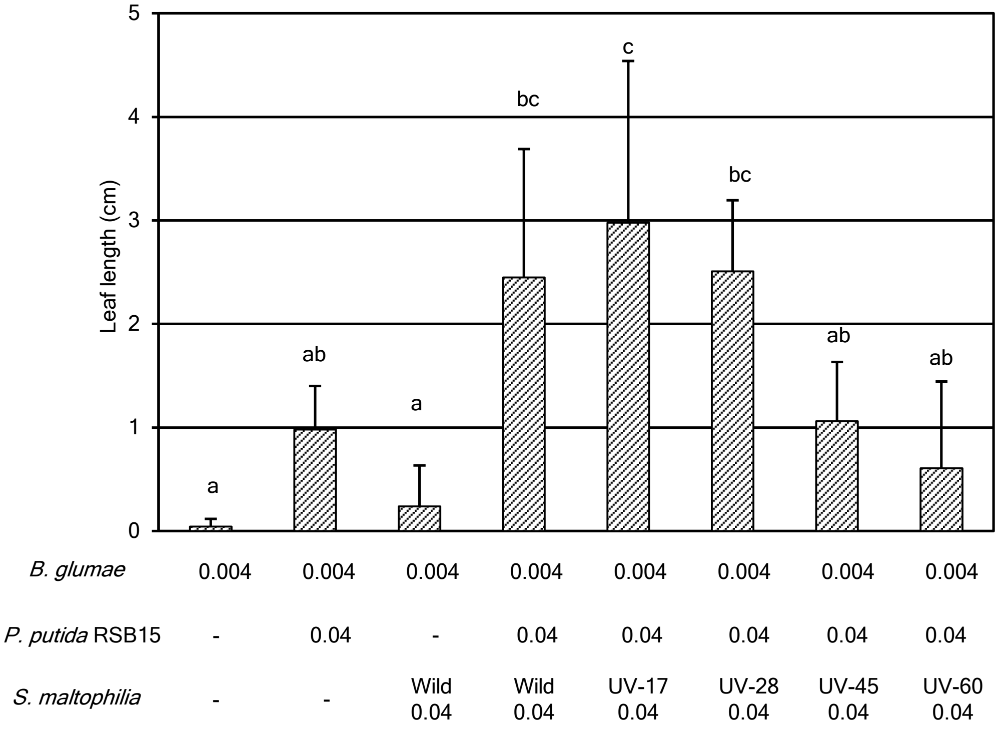

fig. S11 The cooperative effects of *P. putida* RSB15 and *S. maltophilia*RSB2 was dependent on the Biofilm formation ability of RSB2.
The leaf length of the seedlings treated with *P. putida* RSB15 and the biofilm formation mutant of *S. maltophilia* RSB2 derived by UV-mutagenesis were measured at 8 dpi (n=20). Numbers showed optical density of each bacteria in the inoculum. Statistical significance was subsequently determined through a TukeyHSD test (R software). Results are representative of three experiments.

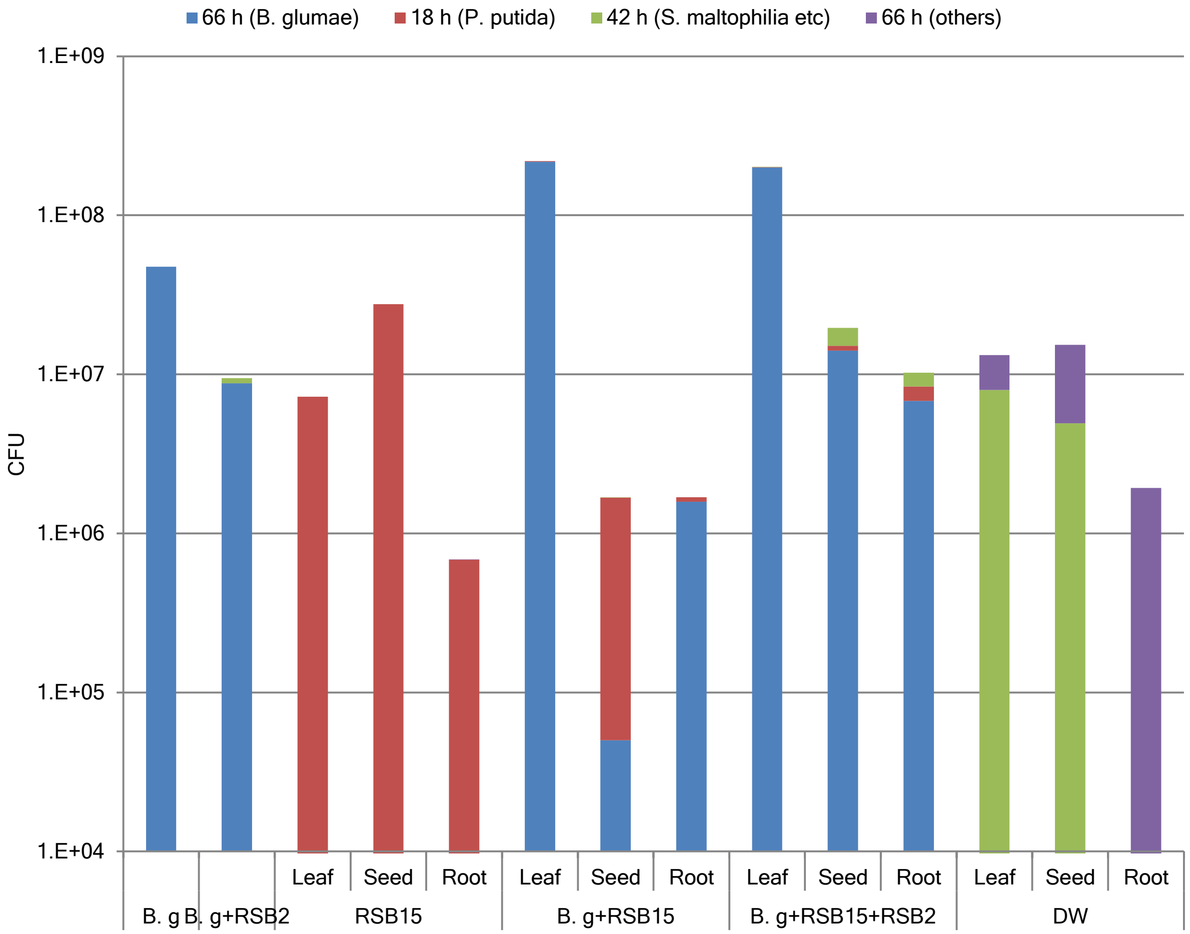

fig. S12 Distribution of the microbes.
The seedlings treated with indicated *B. glumae* (B. g) and RSBs and suspension of 10 dpi seedlings collected by plant parts were diluted and spread on LB plates supplemented with Chrolamphenicol (n=3). The number of colonies that appeared at indicated time points was counted. Results are representative of two experiments.

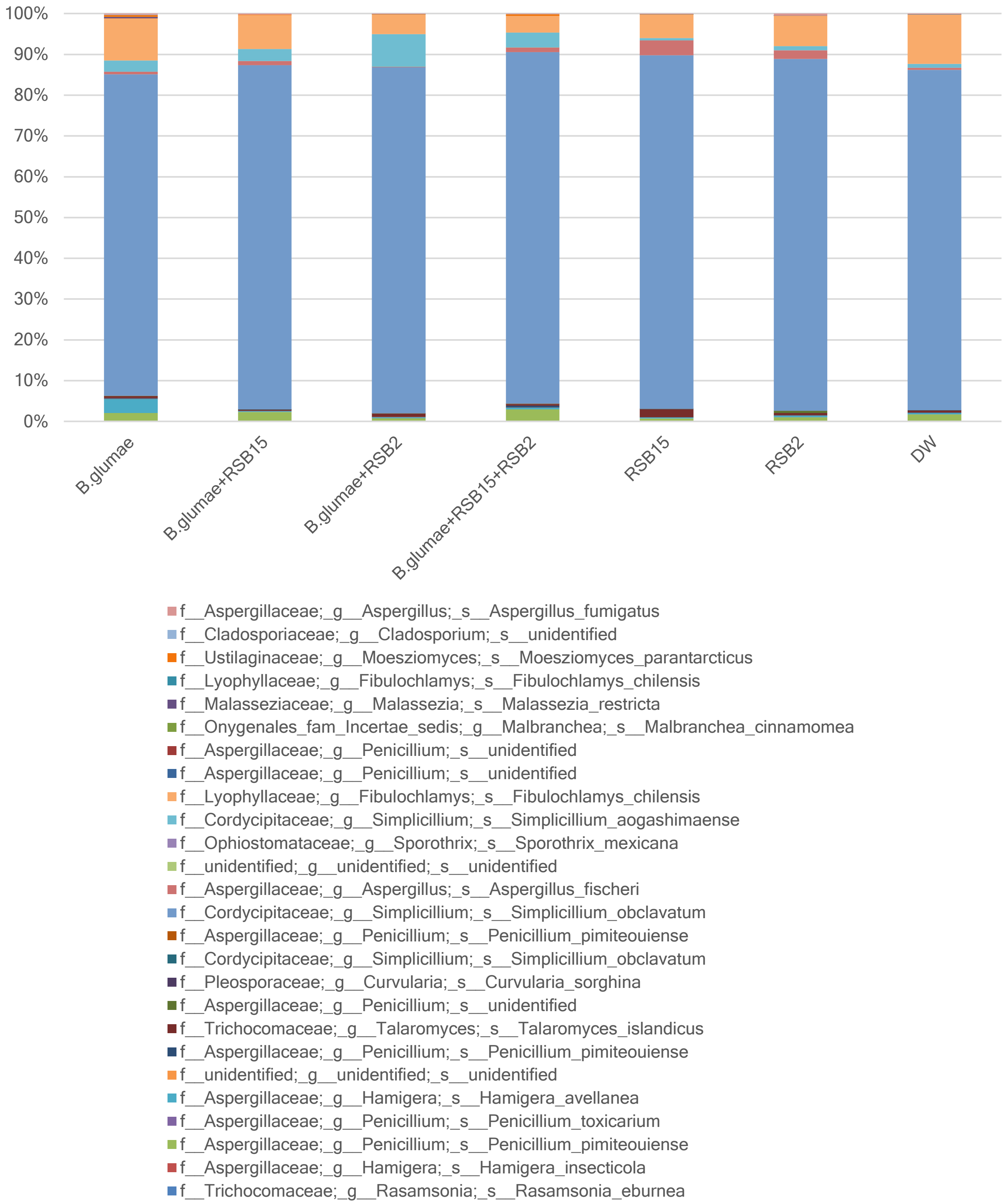

fig. S13 Percentage of OTUs detected in the samples collected from rice seedlings treated with *B. glumae* and RSBs. Results from the analysis of ITS sequences detected by culture-independent evaluation of rice using next generation sequencing with a MiSeq system (n=3).

References

1. Tamura K. and Nei M. Estimation of the number of nucleotide substitutions in the control region of mitochondrial DNA in humans and chimpanzees. *Molecular Biology and Evolution* **10**, 512-526 (1993).
2. Kumar S., Stecher G., Li M., Knyaz C., and Tamura K. MEGA X: Molecular Evolutionary Genetics Analysis across computing platforms. *Molecular Biology and Evolution* **35**, 1547-1549 (2018).
